# Beneficial Effects of* Qigong Wuqinxi* in the Improvement of Health Condition, Prevention, and Treatment of Chronic Diseases: Evidence from a Systematic Review

**DOI:** 10.1155/2018/3235950

**Published:** 2018-10-24

**Authors:** Yu Guo, Mingmin Xu, Zeren Wei, Qingchuan Hu, Yue Chen, Jian Yan, Yulong Wei

**Affiliations:** ^1^School of Acupuncture-Moxibustion and Tuina, Beijing University of Chinese Medicine, Beijing 100029, China; ^2^School of Acupuncture-Moxibustion and Tuina, Chengdu University of Traditional Chinese Medicine, Chengdu 610075, China

## Abstract

**Purpose:**

Qigong is a modality of traditional Chinese mind-body medicine that has been used to prevent and cure ailments, to improve health in China for thousands of years. Wuqinxi, a Chinese traditional Qigong that focuses on mind-body integration, is thought to be an effective exercise in promoting physical and mental wellbeing. Thus, we summarized the evidence and aim to unravel effects of Wuqinxi on health outcomes.

**Methods:**

We performed a systematic review of Wuqinxi studies published in English or Chinese since 1979. Relevant English and Chinese language electronic data bases were used for literature search. The selection of studies, data extraction, and validation were performed independently by two reviewers.

**Results:**

A total of 28 eligible studies were included in this review, among which three are 3 in English and 25 in Chinese. The studies included in this review involve three different experimental designs: (1) 16 RCTs; (2) 2 historical cohort studies; and (3) 10 pretest and posttest studies (PPS). Participants in this review are categorized as either healthy or clinical populations. The results from this systematic review support the notion that Wuqinxi may be effective as an adjunctive rehabilitation method for improving psychological and physiological wellbeing among different age of healthy populations in addition to alleviating and treating diseases among various clinical populations.

**Conclusion:**

The results indicated that Wuqinxi has been thought to be beneficial to improve health and treat chronic diseases. However, the methodological problems in the majority of included studies make it difficult to draw firm conclusive statements. More methodologically rigorous designed large-scale RCTs with a long-term follow-up assessment should be further conducted to examine the effects of Wuqixi on health-related parameters and disease-specific measures in different health conditions. This systematic review lends insight for future studies on Wuqinxi and its potential application in preventive and rehabilitation medicine.

## 1. Introduction

Qigong, as one of the essential elements of traditional Chinese mind-body exercise, has been used to improve physical and psychological health and combat diseases in China for thousands of years [1–3].

*Qi* denotes vital life-energy that flows in energy channels (meridian system) of body, and* Gong* means practice or skill [4, 5]. The characteristic of Qigong is self-directed, and the basic components of Qigong include concentration, relaxation, meditation, rhythmic breathing regulation, body posture, and gentle movements [1, 6–8]. In the current definitions of Qigong [9],* Qigong is the skill of body-mind exercise that integrates the three adjustments of body, breath, and mind into “one”.*

Wuqinxi, also called five-animal exercises, is considered as one of the most widely practiced forms of traditional Chinese Qigong that developed from the I-Ching philosophy and Traditional Chinese Medicine theory [10, 11]. Wuqinxi was created by Chinese well-known physician Huatuo in Donghan Dynasty. According to the ancient “*Daoyin*” and “*Tuna*” (Ancient breathing exercise of Taoism), Huatuo carefully observed the activity characteristics of 5 animals, bear, tiger, ape, deer, and bird, and composed the set of actions integrated with the combination of human body functions and the biological clock [12, 13]. Compared with conventional exercise modalities (e.g., resistance training, muscular endurance training, and strength training), Wuqinxi is characterized by interplay between symmetrical physical postures and movements, breathing control, a meditative state of mind, and mental focus in a harmonious manner [10, 11, 14]. According to the Traditional Chinese Medicine theory,* Qi* is the conceptual foundation in acupuncture, herbal medicine, and Chinese physical therapy. It is considered to be a ubiquitous resource of nature that sustains human wellbeing and assists in healing disease as well as having fundamental influence on all life and even the orderly function of celestial mechanics and the laws of physics [15]. Qigong is based on the theory that body is a small universe where* Qi* circulates and illness or injury disturbs the harmony of vital energy circulation [5, 8, 16]. Wuqinxi typically gains control over* Qi* based on integrating the three adjustments of body, breath, and mind into “*one*” [10, 11, 14, 17] and it aims to achieve a harmonious flow of* Qi*, blood, and fluid throughout the body by long-term practicing to relieve pathological stagnation and regulate the functional activities of meridians and visceral organs [17–19]. With regular practice and rehearsal of the structured postures or movements, as well as concentration on mind and breath, practitioners can achieve an efficiency of “body relaxation and mind calm” and* Tian Ren He Yi* (the theory that mankind is an integral part of nature) so as to experience mood stabilization and improved strength and fitness [17–21]. Convenience and popularity are another feature of Wuqinxi; Wuqinxi exercise does not depend on place, exercise equipment, sex, or different age levels [14, 17, 18].

In addition, Wuqinxi can be comparatively easy to learn with less physical and cognitive demands; therefore, it is also suitable for populations with physical or cognitive impairments [10, 18, 19, 22, 23].

Currently, the most frequently used Qigong Wuqinxi version includes ten movements (commonly two movements for each of the five animals): (1) tiger exercise (including raising the tiger's paws and tiger seizing the prey); (2) deer exercise (including colliding with the antlers and running like a deer); (3) bear exercise (including wobbling body like a bear and rubbing abdomen like a bear ); (4) ape exercise (including being on the alert like a monkey and plunking fruit like a monkey); (5) bird exercise (including stretching upward like a bird and flying like a bird) [9]. Generally speaking, this style may be practiced in its entirety, or learners may choose specific sections to practice. The intensity and duration are also not fixed, making this quite a flexible exercise. It is best to practice until the body warms up and moderate sweating occurs [9, 17, 18]. Evidence arising from reviews and meta-analysis studies has indicated that Wuqinxi is thought to be beneficial for health across different populations [11, 24–36]; for example, current studies have suggested that Wuqinxi appears to have substantive beneficial adjustment on nervous system, hematological system, respiratory system, alimentary system, immune system, and locomotor system. And then an increasing number of studies have documented the effects of Wuqinxi on improving physical and mental health, treating, and curing a variety of chronic diseases including hyperlipidemia, hypertension, knee osteoarthritis, and osteoporosis. Thus researchers have a responsibility to introduce Wuqinxi to the world so that more people are aware of its therapeutic value and may be motivated to engage in Wuqinxi for health. Moreover, despite the growing number of studies assessing the effects of Wuqinxi for health, the absence of critically appraised evidence continues to exist, leaving little clarity for evidence-based clinical practice. The following questions are inconclusive: (1) how exactly is the effectiveness of stand-alone intervention, Wuqinxi for health; (2) how long and how often is Wuqinxi recommended to practice at least. Considering the substantial number of studies produced over the last decades on the health benefits of practicing Wuqinxi, it is valuable for the research community to have access to a comprehensive review and summary of study results. This first English review study taking Wuqingxi as an example aims to fill the gap in the literature and evaluate up-to-date evidence of Wuqinxi for health in English or Chinese language studies as well as better understand current trends in Wuqinxi for different health benefits, which may provide insight for the need of investigators and future research.

## 2. Methods

### 2.1. Data Sources

Five well-respected electronic databases (PubMed, Cochrane Library, Web of Science, Embase, and Medline) were used for English literature search to maximize the potentially relevant literature search; the following keywords were used by review authors: ‘‘Wuqinxi”,‘‘Wuqinxi Qigong”, ‘‘Five-animal exercise”, ‘‘Five-animal boxing”, ‘‘Five animals play”, ‘‘Five animals frolic”, ‘‘Five poultry drills”. The WanFang Database and Chinese National Knowledge Information Database (CNKI) were also adopted to search Chinese literature by using the keyword “五*禽戏*” or “五*禽戲*” (Wuqinxi). Following electronic search completion, identified records were initially screened for eligibility based on title and abstract. Reference lists of systematic reviews and their original studies were manually searched for additional studies in related areas. Articles were finally selected based on the analysis of the full text. Two reviewers, Y. Guo and MM. Xu, independently applied the inclusion criteria. A review (XMM) was responsible for contacting with Chinese Qigong experts in order to gain a comprehensive understanding of Wuqinxi.

### 2.2. Study Selection

The studies were included in this review if they met the following criteria: (1) a peer-reviewed study or a doctoral dissertation (because a doctoral dissertation typically undergoes a rigorous peer-review process through the supervision of three to five committee members) published in English or Chinese; (2) retrievable full text articles; (3) Wuqinxi as the primary intervention in the studies; (4) a minimum of one health-related outcome measure was reported in the peer-reviewed study such as physical fitness (e.g., balance, flexibility, muscular strength, muscular endurance, aerobic ability, or mobility), psychological domain (e.g., pain, stiffness, fatigue, depression, anxiety, or self-efficacy), physiological domain (e.g., blood pressure, heart rate, bone mineral density (BMD), and pulmonary function), cognitive function, or quality of life (QOL) (the review authors extracted the most commonly reported outcomes); (5) participants who must be adult men and women aged 18 and over (with no upper age limit).

To gain a comprehensive understanding regarding the effects of Wuqinxi in both healthy and clinical populations, the study designs could be a randomized controlled trial (RCT), nonrandomized controlled trail (NRCT), controlled trial, uncontrolled trial, pretest and posttest study (PPS), cohort study, or cross-sectional/observational study. In addition, in order to ensure the quality of review, Chinese studies must be published in the Chinese core journals. The year range of publications between 1979 (first article introducing Wuqinxi) and the search date was considered. Studies that did not meet the abovementioned criteria were excluded (e.g., case reports, letters, book reviews, meta-analyses, literature reviews, magazine editorials, commentaries, master's dissertation, and conference proceeding). Two review authors (GY and XMM) independently selected eligible studies according to the same eligibility criteria. Any disagreements between the two reviews were resolved through discussion. Consulting a third author (HQC) also reviewed the study and facilitated the decision when a disagreement persisted.

### 2.3. Data Extraction

Two review authors (GY and XMM) independently extracted the characteristics of each included study onto predesigned excel forms based on a predetermined data of basic information (name of author, year of publication, country of study, study design/blinding, sample size, mean and standard deviation for age, sex composition, number of participants in study groups, relevant outcomes measured, results, adverse events, conclusion, and follow-up assessment) and detailed information relating to the interventions (frequency and duration and comparison details). A third party (HQC) appeared and had dealt with disagreement between the two reviewers. Any discrepancies were discussed until consensus was achieved.

### 2.4. Selection Process

**Figure 1:**
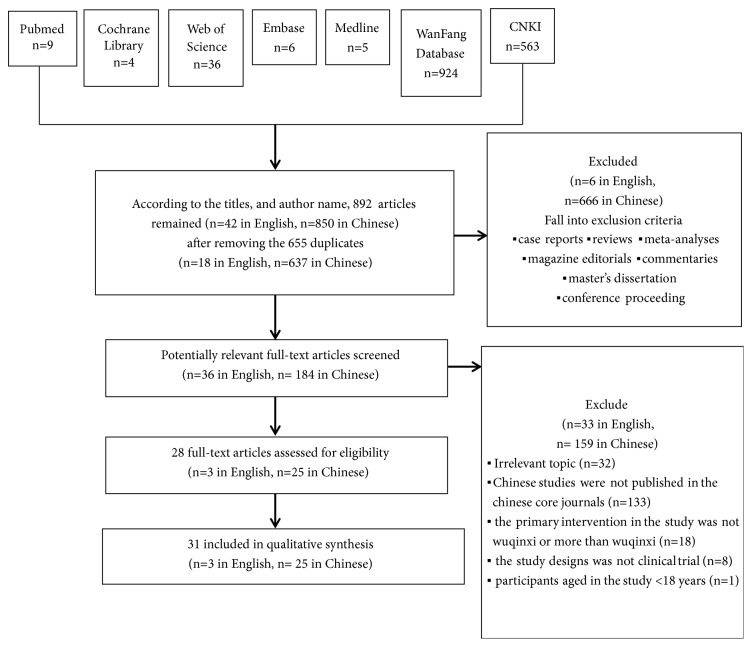
Flowchart displaying the process of literature search.

The literature search acquired 1547 literature items (n =60 in English, n =1487 in Chinese), among which duplicated works and those that fell into the exclusion criteria were ruled out. The second step was to remove those which did not meet the inclusion criteria, which resulted in a final 28 publications (n =3 in English, n =25 in Chinese) selected for further review (Figure 1 displaying the process of literature search).

## 3. Results

The earliest studies regarding the influences of Wuqinxi for health benefits were published in 2003 [55, 56], followed by two studies published in 2004 [53, 54], and the earliest study regarding the effects of Wuqinxi on the immunologic function in the subjects was published in 2005 [52]. And from the year 2006 to 2008, seven articles about the improvement of Wuqinxi for health in middle-aged and elderly people were published in the Chinese core journals in succession [45–51]. The earliest study regarding the treatment of Wuqinxi for patients was published in 2009 [61]. And the earliest two English language studies regarding the influences of Wuqinxi for healthy people or obese old people were published in 2011 [43, 44]. The first doctoral dissertation was published in 2012 [60]; at the same year, a Chinese language study about the treatment of Wuqinxi on cognitive function of patients with metabolic syndrome [23] and four Chinese language studies regarding the influence of Wuqinxi for health benefits were published [39–42]. Most recent Chinese language or English language studies were published between 2013 and 2018 [10, 22, 37, 38, 57–59]. A total of 28 studies were found to meet the inclusion criteria [10, 22, 23, 37–61]. The interrater reliability between the two reviews was 96.4% for selecting eligible studies. The studies included in this review involve three different experimental designs: (1) 16 RCTs [10, 22, 23, 38–40, 45–48, 51, 57–61]; (2) 2 historical cohort studies [37, 42]; and (3) 10 pretest and posttest studies (PPS) [41, 43, 44, 49, 50, 52–56]. Study participants in this review are categorized as either healthy or clinical populations. More specifically, the healthy populations were college students [39], older adults [22, 41, 43, 44, 47, 51], and middle-aged and elderly adults [10, 37, 38, 40, 42, 45, 46, 48–50, 52, 56, 60]. The clinical populations were patients with mild depression disorder [57], osteoporosis (OA) [59], knee osteoarthritis (KOA) [58], metabolic syndrome (MS) [23], and dyslipidemia [61]. The health-related parameters (cognitive function, immunologic function, physical, psychological, and physiological parameters) are reported in detail under current trend in Wuqinxi for healthy populations section. Type of special population (mild depression disorder, osteoporosis (OA), knee osteoarthritis (KOA), metabolic syndrome (MS), and dyslipidemia) is reported in detail under current trends in Wuqinxi for clinical populations section. Characteristics of eligible studies are presented in Tables 1 and 2.

### 3.1. Current Trend in Wuqinxi for Healthy Populations

#### 3.1.1. Cognitive Function

Three studies examined the effects of entire Wuqinxi exercise on cognitive function in middle-aged and elderly people. Chen et al. [50] examined the effects of Wuqinxi on attention concentration ability. One-hundred middle-aged and elderly volunteers were arranged to participate in seven 45 min Wuqinxi sessions weekly for 24 weeks. It was observed that exercising Wuqinxi effectively improved the middle-aged and elderly people's attention concentration ability. Besides, after the 8-week intervention period (seven times weekly), results in a PPS by Wu et al. [56] also showed beneficial improvement of Wuqinxi on attention concentration ability. A historical cohort study by Liang [37] used simplified version of graphic reasoning (Raven) test and cancellation test to evaluate the cognitive function of middle-aged and elderly people with exercising Wuqinxi in four 30 min Wuqinxi sessions weekly for at least 2 years. The results further indicated that Wuqinxi had a better effect on cognitive function of middle-aged and elderly people.

#### 3.1.2. Immunologic Function

Five studies examined the regulating effects of entire Wuqinxi exercise on immunologic function in middle-aged and elderly people. Yu et al. [52] conducted the earliest PPS examining the influence of Wuqinxi on plasma NK cell activity in middle-aged and elderly people. Fifty-three volunteers participated in a Wuqinxi program which is at least four 26~39 min sessions weekly for 12 weeks. Result indicated that after 12 weeks of Wuqinxi intervention, the plasma NK cell activity was obviously higher than that before exercise; the authors gave the view that Wuqinxi may be had better effect on regulating the immunologic balance of middle-aged and elderly people.

Based on that, two other RCT studies by Yu et al. [45, 48] investigated the improvement effects of Wuqinxi on plasma NK cell activity and peripheral blood T-cell subsets (CD_4_^+^, CD_8_^+^, CD_3_^+^, and the ratio of CD_4_^+^/ CD_8_^+^) compared with control group after intervention period. The first RCT [45] investigated the effect of Wuqinxi on plasma NK cell activity in middle-aged and elderly people. This study involved a 24-week intervention period for a Wuqinxi group (at least four 45 min training sessions weekly for Wuqinxi) and a control group (unaltered daily lifestyle). Results indicated that Wuqinxi was thought to be effective in increasing the activity of plasma NK cells and adjusting the balance of middle-aged and elderly people's immune ability. The second RCT [48] included 100 middle-aged and elderly people in which they were randomly assigned to either a Wuqinxi group (at least four 45 min Wuqinxi sessions weekly) or a control group (unaltered daily lifestyle). Results indicated that 24 weeks of Wuqinxi were thought to have a positive effect on regulating the peripheral blood T lymphocytes of middle-aged and elderly people. In addition, Wu et al. [56] subsequently conducted a PPS which examined the effects of a 12-week Wuqinxi program using the same outcome measures. And results also indicated that the practicing Wuqinxi may affect the distribution of the outer subgroup of T-cell of the middle-aged and elderly people, and it was thought to be good for the improvement of the immunity of middle-aged and elderly people. A historical cohort study by Liang [37] indicated that, compared with control group (unaltered lifestyle), CD_3_^+^, CD_4_^+^, and CD_8_^+^, natural killer (NK) cell in Wuqinxi group (four 30 min training sessions weekly for at least 2 years) was significantly higher than those in the control group. This historical cohort study author also thought that Wuqinxi had effect on regulating and improving body immunity of middle-aged and elderly people.

#### 3.1.3. Physical and Psychological Parameters

Yu et al. [55] conducted a PPS regarding the influences of entire Wuqinxi exercise for physical and psychological parameters, in which 85 healthy volunteers were arranged to practice seven Wuqinxi sessions weekly for 8 weeks. Outcome measures include body shape index, physical function index, physical quality index, and Self-Rated Health Measurement Scale Version (SRHMS) that was used to evaluate the psychological wellbeing. From the results we can see that Wuqinxi showed a positive effect on the body shape, physical function, and physical quality of the volunteers and also had a better effect on improving and adjusting the mental state of the volunteers. A PPS by Cui et al. [53] recruited 200 healthy female middle-aged and elderly volunteers in which they were informed to exercise three 60 min entire Wuqinxi training sessions weekly for 12 weeks. Results showed that female middle-aged and elderly volunteers' blood pressure, pulse, waist/hip ratio, grip strength, and vital capacity have had obvious improvement after the exercise of Wuqinxi three months later; at the same time, an evident effect on mentality follows.

#### 3.1.4. Physical Parameters

Yu et al. [54] subsequently carried out another PPS to further investigate the effects of a 12-week entire Wuqinxi intervention (three 60 min sessions weekly) in female middle-aged and elderly people. They reported that female middle-aged and elderly volunteers' waistline, waist/hip ratio, systolic pressure, and diastolic pressure have had obvious decrease after three months of exercising Wuqinxi; at the same time, there were evident improvement effects on vital capacity and grip strength after exercise. And so they all agreed that female middle-aged and elderly who does Wuqinxi exercise may achieve satisfaction effects on body shape, physical function, and physical quality. From the aspect of the improvement effects of Wuqinxi on the function of the lumbosacral multifidus, a historical cohort study was conducted by Bai et al. [42] to compare the effects of entire Wuqinxi exercise with aerobic exercise (walking) on the average surface electromyography (ASEMG) in the process of flexion and extension was recorded and analyzed by Biovision surface electromyography test system software in the healthy middle-aged and elderly people. Both groups received the same duration and frequency of training (five training times weekly for 1 year). Results indicated that the Wuqinxi was thought to have positive improvement on the function of the lumbosacral multifidus in the middle-aged and elderly so as to reduce low back pain. Zhang et al. [10] subsequently conducted an RCT which investigated the effects of 1-year entire Wuqinxi training using the same outcome measures. All male volunteers were assigned into either a Wuqinxi group (15 participants who attended five training times weekly for 1 year) or a control group (15 participants who attended five 30 min walking training times weekly for 1 year). The authors in this study believed that Wuqinxi was beneficial to the function of the lumbosacral multifidus.

#### 3.1.5. Psychological Parameters

From the aspect of emphasizing improving the psychological effects of entire Wuqinxi exercise in people, Wu et al. [56] investigated the preventive psychological effects of Wuqinxi in healthy people, which were measured by Self-Rated Health Measurement Scale Version (SRHMS) and Perceived-Exercise-Effect Rating Scale (PEERS). After the 8-week intervention period (seven times weekly), the results from this PPS suggested that Wuqinxi was thought to have certain effects on stabilizing mood, regulating psychological state, and improving the quality of life. In addition, two studies deeply explored the effects of entire Wuqinxi exercise on mental adjustment measured by psychological self-assessment scales. Among the two studies, a historical cohort study by Liang [37] used Self-Report Symptom Inventory (SCL-90) and BFS mood scale to observe the psychological effects of Wuqinxi in middle-aged and elderly people. From the results Liang believed that Wuqinxi was thought to be better effect on improving the activity and pleasurable mood. An RCT by Chang et al. [38] examined the psychological effects of different Qigong exercises in middle-aged and elderly people. All eligible healthy middle-aged and elderly people were randomly allocated to either a Wuqinxi group (five Wuqinxi 60 min sessions weekly for 12 weeks) or a Yijinjing group (five Yijinjing 60 min sessions weekly for 12 weeks) or a Liuzijue group (five Liuzijue 60-min sessions weekly for 12 weeks) or a Baduanjin group (five Baduanjin 60 min sessions weekly for 12 weeks). Results indicated that four kinds of Qigong exercises showed better health preservation value and had a positive impact on regulating and improving middle-aged and elderly people's mental state. When compared with other groups, Wuqinxi was thought to effectively improve anxiety and self-test health.

#### 3.1.6. Physiological and Biochemical Parameters

An RCT by Yu et al. [40] included 120 middle-aged and elderly volunteers. All middle-aged and elderly volunteers were randomly assigned to either a Wuqinxi group (four entire Wuqinxi sessions weekly for 1 year) or a control group (conventional exercise). Significant improvements in multiple outcome measures (physiological function index and physical quality index, and biochemical index) were observed in the Wuqinxi group, but not in the control group. Meanwhile, an RCT by Qin [39] included 20 healthy college students in which they were randomly assigned to either a Wuqinxi group (six 60 min Wuqinxi sessions weekly) or a control group (unaltered daily lifestyle). The purpose of the study was to determine whether a 20-week entire Wuqinxi program was beneficial for cardiac function and vascular function. Results indicated that, when compared with the control group, the Wuqinxi group showed significant increase in SV, CI, VPE, HDL, and remarkable decreased in HR, MSP, MDP, MAP, and TR. We can, therefore, come to the conclusion that Wuqinxi maybe had a positive impact on cardiac function and vascular function in healthy college students. From the point of lipid metabolism in middle-aged and elderly people, an RCT by Yu [46] discussed how 24-week entire Wuqinxi training affected lipid metabolism capability in middle-aged and elderly people. All eligible participants were assigned into either a Wuqinxi group (50 participants who attended at least four 45 min training sessions for 6 months) or a control group (unaltered lifestyle). Total cholesterol (TC), triglyceride (TG), high density lipoprotein cholesterol (HDL-C), and low density lipoprotein cholesterol (LDL-C) were measured to evaluate the effects of Wuqinxi on lipid metabolism. From the results we can get that six months of Wuqinxi showed a certain regulation effect on improving lipid metabolism in the middle-aged and elderly people. Two RCTs by Zhu [47, 51] were conducted to determine whether the entire Wuqinxi exercise was beneficial for physical function and antioxidant capacity in female elderly people. The first RCT [41] used malondialdehyde (MDA), superoxide dismutase (SOD), estradiol (E2), cardiac functional capacity (FC) test, blood pressure (BP), heart rate (HR), vital capacity (VC), reaction time, exercise time, time for closed eyes one-foot balance to compare physical function, and antioxidant capacity between Wuqinxi group and control group (unaltered lifestyle). Wuqinxi group received training about 45-min session daily for 16 weeks. After the intervention period, we can draw a conclusion that Wuqinxi was thought to effectively slow the process of ageing by increasing serum SOD activity, delaying the free radical injury increased with ageing, improving the level of sexual hormone, and enhancing feeling and balance ability. Moreover, the second RCT [51] included 75 female elderly people in which they were assigned to either a Wuqinxi group (45 participants attending 45 min session daily for 16 weeks) or a control group receiving no intervention and keeping unaltered lifestyle. Results suggested that it may be possible for Wuqinxi to improve the activity of peripheral blood SOD, delay the damage of free radical, increase the level of sex hormone, and enhance the ability of nerve reaction and balance in the female elderly people. On the basis of foregoing studies, three studies were conducted to explore the regulation effects of entire Wuqinxi exercise in older people from the aspect of blood antioxidant enzymes activities or lipid peroxidation or intestine probiotics count. Sang [43] conducted a PPS examining the effects of Wuqinxi on serum antioxidant enzymes activities, lipid peroxidation, and intestine probiotics count in obese older people. 55 obese older people who met the inclusion criteria participated in a Wuqinxi program of 120 min session daily lasting for 48 weeks. The results provided proof that 48 weeks of Wuqinx were thought to enhance serum antioxidant enzymes activities, lipid peroxidation, and intestine probiotics count in obese older people. A PPS by Chen [44] investigated the preventive effects of Wuqinxi on blood lipid levels and antioxidant enzyme activities in 64 healthy older people who received 60 min Wuqinxi session daily for 30 days. Results showed that 30 days of Wuqinxi maybe had a positive impact for older people about decreasing blood lipids levels and oxidative injury so that it is useful for older peoples' health. Besides, a PPS by Duan [41] examined the effects of Wuqinxi on antioxidant capacity and intestine lactobacillus. 60 elderly volunteers were arranged to practice in six 45 min Wuqinxi sessions weekly for 16 weeks. From the results, we can get that Wuqinxi showed effects of producing SOD, GSH, and reducing substance to enhance antioxidant capacity and increasing quantity of intestine lactobacillus, which may be the physiological mechanism that Wuqinxi had effects on improving health of elder people.

### 3.2. Current Trends in Wuqinxi for Clinical Populations

#### 3.2.1. Mild Depression Disorder

Cheng et al. [57] conducted an RCT in which 30 college students with mild depression disorder and 28 healthy college students were randomly assigned to either a Wuqinxi group (including 1 week of learning phase, three 40~60-min entire Wuqinxi training sessions weekly for 12 weeks of practicing phases) or a control group (unaltered lifestyle) according to 1:1 ratio. Outcome measures included BECK depression self-reported questionnaire (BDI) and Hamilton depression rating scale (HAMD). The metabolic parameters of 1H- MRS in the prefrontal cortex and hippocampus (NAA/Cr, Cho/Cr, NAA/Cho, Cho/NAA) were used to explore the potential mechanism of how Wuqinxi affects mental and emotional states. From the results we can arrive at a conclusion that exercising Wuqinxi was thought to reduce depression scale scores in college students with mild depression by improving the expression of metabolic indexes (NAA/Cr and Cho/Cr values) in the prefrontal cortex and the hippocampus.

#### 3.2.2. Osteoporosis (OA)

Two RCTs by Shen et al. [22, 59] were conducted to explore whether entire Wuqinxi was beneficial for elderly patients with senile osteoporosis. The first RCT study [59] included 200 patients with senile osteoporosis. All patients were randomly assigned to either a Wuqinxi group (six 45 min sessions weekly for 24 weeks) or a control group (treating with ibuprofen sustained release capsules (Fenbid) and calcium carbonate and vitamin D3 tablets, 1 tablet/time, 2 times/day). After the 24-week intervention, BMD of the lumbar vertebrae in Wuqinxi group was higher than that in control group significantly and the low back pain scores in the Wuqinxi group were lower than that in control group remarkably. The authors in this study viewed that practicing Wuqinxi was thought to be an effective way to prevent and cure primary osteoporosis and can be applied in communities. In the next year, Shen et al. [22] carried out another deeply RCT study regarding the influence of Wuqinxi for elderly patients with senile osteoporosis. The purpose was to compare the effects of Wuqinxi with drug therapy on bone metabolism index (serum osteocalcin (BGP), alkaline phosphatase (ALP), level of pyridinoline (PYD)) and low back pain score of visual analogue scale (VAS) in 200 elderly patients with senile osteoporosis. Wuqinxi group received six 45 min Wuqinxi training sessions weekly for 24 weeks, whereas control group was treated with ibuprofen sustained release capsules (Fenbid) and calcium carbonate and vitamin D3 tablets (1 tablet/time, 2 times/day). After the intervention, the author was convinced that practicing Wuqinxi was positive to the bone metabolism of the senile osteoporosis patients and can effectively relieve and improve the low back pain syndromes of the senile osteoporosis patients. To a certain degree, this exercise may also increase bone formation and decrease bone resorption.

#### 3.2.3. Knee Osteoarthritis (KOA)

Two studies examined the therapeutic benefits of entire Wuqinxi exercise for female middle-aged and elderly patients with knee osteoarthritis. A doctoral dissertation by Tian [60] included 40 female middle-aged and elderly patients with knee osteoarthritis and randomized them into either a Wuqinxi group (six 60 min training sessions weekly for 24 weeks) or a KOA control group; in addition, this RCT study also recruited 20 healthy middle-aged and elderly females who were assigned to healthy control group. All of the control groups received no treatment and performed to keep unaltered lifestyle. Compared with the control group before and after intervention, the author came to conclusion that it was thought to be good for middle-aged and elderly patients with KOA to practice Wuqinxi in a long term, which can effectively improve the proprioceptive function, dynamic and static balance of knee and reduced their risk of falls. Tu et al. [58] subsequently conducted another RCT which recruited 40 female patients with knee osteoarthritis to investigate the improvements of Wuqinxi on the peak torque (PT) and total work (TW) of the affected knee obtained by the isokinetic testing system and the level of pain, stiffness, dysfunction measured by The Western Ontario, and McMaster Universities (WOMAC) Osteoarthritis Index Scale. All female patients with knee osteoarthritis were randomly assigned to either a Qigong Wuqinxi group or a Qigong Zhanzhuang (standing exercise) group. But two groups received the different duration and frequency of training (Wuqinxi: six 60 min training sessions weekly for 16 weeks and Zhanzhuang: 10 mins/session, 3 sessions/time/day, 6 times/week, 16 weeks). This study made the conclusion that both Wuqinxi exercise and Zhanzhuang exercise had effect on improving the quadriceps strength of female patients with KOA and reducing the effect of pain, stiffness, dysfunction and so on. Wuqinxi exercise was thought to have a smooth and comprehensive influence on the knee's flexor and extensor strength of female patients with KOA when compared with Zhanzhuang exercise and it was helpful for relieving pain and reducing dysfunction.

#### 3.2.4. Metabolic Syndrome (MS)

An RCT by Liu et al. [23] examined the influences of entire Wuqinxi exercise on elderly patients with metabolic syndrome. This study included 40 elderly patients with metabolic syndrome in which they were randomly assigned to either a Wuqinxi group (including 1 week of learning phase, six 60 min entire Wuqinxi training sessions weekly for 24 weeks of practicing phases) or a control group (unaltered lifestyle). The vascular risk factors including body mass index (BMI), fasting blood gluco (FBG), total cholesterol (TC), triglyceride (TG), high density lipoprotein cholesterol (HDL-C), and low density lipoprotein cholesterol (LDL-C) and the neuropsychological index including minimental state examination (MMSE), Montreal cognitive assessment (MOCA), verbal fluency test (VFT), trail making test (TMT), and Hamilton depression rating scale (HAMD) were used as outcome measures to evaluate the improvement of Wuqinxi. The study findings supported the notion that the vascular risk factors and the cognitive function of elderly patients with metabolic syndrome was improved after Wuqinxi training.

#### 3.2.5. Dyslipidemia

Li et al. [61] conducted an RCT in which 66 patients with dyslipidemia were randomly assigned to either a Wuqinxi group (30 min entire Wuqinxi training session daily for 16 weeks) or a control group (aerobic exercise (jogging)). The purpose of this study was to investigate the effect of Wuqinxi on lipids metabolism in patients with dyslipidemia. The results analysis indicated that TC, TG, and LDL-C of the Wuqinxi group declined remarkably and HDL-C of the Wuqinxi group rose evidently after 16 weeks of Wuqinxi training. It was worth noting that better improvements in TC, TG, and LDL-C were shown in the Wuqinxi group, whereas these positive changes were not observed in the control group. In addition, the rate of reaching standard in the Wuqinxi group was higher than that in the control group. In this study, the authors brought forth conclusion that Wuqinxi was thought to be effective for patients with dyslipidemia.

## 4. Discussion

The “Qigong Wuqinxi” values the regulation of the breathing and aspiration, requiring an enhancement to the breath depth and cycle, and trying to reach a “fine, deep, long and balance” target as far as possible [11]. Also, the “Qigong Wuqinxi” is an all-round exercise of human body, including bending forward, backward and sideward, and twisting. It exerts a good stretching role in the chest, waist, leg muscle, and ligaments and hence can enlarge the movement range of the spinal column, waist and legs [10, 42, 58, 60]. Therefore, the “Qigong Wuqinxi” can combine the body movements, the breathing and aspiration, and the psychological adjustment together harmoniously. It can not only stretch human body and ease joints, but also have an adjustment effect on the subhealth of human body. Regarding the role of Wuqinxi in the improvement of health, we aimed to provide a systematic review of both the English and Chinese literature to address the efficacy and safety of Wuqinxi in the improvement of psychological and physiological health. In this systematic review, the effects of Wuqinxi on improving health-related parameters in both healthy populations (e.g., college students, middle-aged and elderly adults, male adults, and older adults), and various clinical populations (e.g., mild depression disorder, osteoporosis, knee osteoarthritis, metabolic syndrome, and dyslipidemia) were examined comprehensively. To summarize, this systematic review involved 16 RCTs, 2 historical cohort studies, and 10 PPSs. The focus on cognitive function, immunologic function, physical, psychological, physiological, and biochemical parameters in healthy populations, and clinical populations with different diseases. In accordance with the results of the experimental research, two PPSs [50, 56] and a historical cohort study [37] both suggested favorable effects of Wuqinxi on the improvement of cognitive function (including attention concentration ability, intelligence quotient, and memory ability) among middle-aged and elderly people. Specifically, two RCTs [45, 48] and two PPSs [49, 52] as well as a historical cohort study [49] believed that Wuqinxi showed benign regulation on immunologic function (including plasma natural killer (NK) cell activity, peripheral blood T-cell subsets (CD_4_^+^, CD_8_^+^, CD_3_^+^ and the ratio of CD_4_^+^/ CD_8_^+^)) of middle-aged and elderly people. In the physical, psychological, physiological, and biochemical parameters, seven RCTs [10, 38–40, 46, 47, 51], and seven PPSs [41, 43, 44, 53–56], two historical cohort studies [37, 42] suggested a beneficial effects of Wuqinxi on the improvement of body shape index, physical function index, physical quality index, biochemical index, psychological wellbeing, and lipid metabolism capability, serum antioxidant enzymes activities, function of the lumbosacral multifidus, and intestine probiotics count. The other clinical trials [22, 23, 57–61] indicate that the treatment of Wuqinxi on diseases is still very much underinvestigated with the scarcity of scientific studies in the field. Nonetheless, the available evidence seems to suggest that Wuqinxi was thought to have great potential to become an integral part for enhancing health and promoting overall quality of life among patients with different diseases. For instance, an RCT [57] in our review found that Wuqinxi was thought to reduce depressive symptoms in college students with mild depression by improving the expression of metabolic indexes in the prefrontal cortex and the hippocampus. With regard to objective outcomes of osteoporosis and knee osteoarthritis, two RCTs [22, 59] reported that practicing Wuqinxi was positive to the bone metabolism and it can effectively relieve and improve the low back pain syndromes of the senile osteoporosis patients. The other two RCTs [58, 60] concluded that practicing Wuqinxi in a long term may be effectively improved the proprioceptive function, dynamic and static balance, and flexor and extensor strength of knee, relieving pain, and reducing risk of falls and dysfunction, while two RCTs [23, 61] observed that Wuqinxi was thought to help to significantly reduce the vascular risk factors of elderly patients with metabolic syndrome and effectively improved TC, TG, and LDL-C of patients with dyslipidemia.

To date, trials of Wuqinxi particularly focusing on physical and psychological health from healthy populations and clinical populations are still limited due to there being no large-scale RCT study in the literature. Based on currently available literature, interpretation and generalization of our results in this review should be treated with caution as there is limited number of included studies and methodological problems inherent in these studies. For instance, some of the selected studies had potential flaws in their designs: lack of randomization, lack of comparable controls who received the same amount of attention, small sample size leading to false negative or unstable results, lack of blinded assessors and statisticians, unclear description for learning and practicing protocol, and lack of monitoring of adherence to practicing at training ground as well as the varied dosage and quality of Wuqinxi exercise, heterogeneous comparison groups, and outcome measures. And none of the included trials mentioned ethical approvals and clinical trial registration. There could be publication bias as studies with positive outcomes are more likely to be published. Therefore, it is difficult to draw a definitive conclusion regarding the beneficial effects of Wuqinxi based on the selected studies. To avoid the abovementioned limitations, we provide the following recommendations for future Wuqinxi studies.

### 4.1. Study Locations

Although great effort was made to avoid location bias during data retrieval, almost all trials identified after comprehensive searches were conducted in mainland China and published in Chinese with positive results favoring Wuqinxi. Notably, a majority of included studies were conducted by the Shanghai University of Sport in China from 2003 to 2008 [45, 46, 48, 49, 53–56]. Because most of existing studies have been done by Chinese scientists with little support and few resources, some studies were even conducted by Qigong practitioners with no support from healthcare professionals. In addition, only three studies regarding effects of Wuqinxi on antioxidant enzymes activities, lipid peroxidation level, intestine probiotics, blood lipid levels, and lumbosacral multifidus reviewed are in English and accessible to the international community. But it is a pity that only one study was not done in China among three English language studies included in this review. On the other hand, among 33 potentially relevant English articles excluded in this review, there are 30 English articles excluded that belong to irrelevant topic; there are 2 English articles excluded because the primary intervention in the study was not Wuqinxi alone, and 1 English articles was excluded because participants' age in this study was less than 18 years. Therefore, we cannot completely rule out potential location bias. Even so, it is worth noting that Wuqinxi has been shown to be as effective as tai chi or Baduanjin when considering prevention of disease and improvement of physical and mental health. It is also important to acknowledge that, compared with the complex movements of tai chi, Wuqinxi can be practiced with less physical and cognitive effort. It appears that the research community is taking the lead to introduce Wuqinxi to the world so that more people are aware of its beneficial effects and are motivated to participate in this type of exercise for health improvements.

### 4.2. Study Participants

The number of study participants in the studies ranged from 30 to 200; however, no study was reported to use power analysis calculations to determine an adequate sample size. This could impact the ability to detect the statistical differences on both healthy and clinical populations. Thus whether the sample sizes met the basic requirements of clinical research in the trials is unknown. It is also unclear if the sample sizes in some studies were large enough to avoid type II error. Therefore, a relatively large number of participants need to be recruited based on power calculation for future studies to confirm the beneficial effects of Wuqinxi, particularly for clinical populations. The process of sample size estimation and power calculation should be clearly described by investigators of future studies as well. On the other hand, although participants in the selected studies satisfied the eligibility criteria before the beginning of the Wuqinxi program, it is worth noting that researchers from one study recruited diverse groups of patients (e.g., diabetes, hypertension, and heart disease) but did not clearly report the number of patients and their disease stages for each specific condition. The interpretation of study results could be inaccurate due to the heterogeneity of the populations; it was not possible to generalize the study results to a specific clinical population. For instance, in an RCT [23], which included elderly patients with metabolic syndrome, participants in the Wuqinxi group demonstrated significant improvements on the vascular risk factors and the neuropsychological index compared with the control group after participating in a Wuqinxi intervention protocol (six 60 min training sessions weekly for 24 weeks). However, the study's results suggestive of beneficial effects from Wuqinxi training cannot be specifically generalized to patients with hypertension or with overweight and / or obesity, or with hyperglycaemia, or with dyslipidemia. In addition, the training intensity (duration and frequency) that is suitable for patients with hypertension may not be suitable for patients with overweight and/or obesity because patients with overweight and/or obesity may only be able to tolerate less strenuous exercises.

Therefore, future studies that include patients with various conditions should report detailed information regarding the condition characteristics of the study participants. At the same time, the lack of information on drop-outs and withdrawals was also problematic, and in the analysis phase, intention-to-treat analysis should be performed in randomized trials. Nevertheless, details of drop-outs and withdrawals were described in 2 RCTs [46, 51] regarding current trend in Wuqinxi for healthy populations and 2 RCTs [22, 59] regarding current trends in Wuqinxi for clinical populations. None of included studies adopted intention-to-treat analysis, which might have led to the exclusion of some particular participants, causing attrition biases which in turn weaken the quality of studies.

### 4.3. Wuqinxi Intervention

The Wuqinxi intervention of all included studies was entire five-animal exercises; however, there was a great disparity of dosage and intensity of Wuqinxi across the examination of the included studies. For example, “dosage” (i.e., frequency, duration and level of intensity, estimate of aerobic level, or metabolic equivalents) may be important in whether or not benefits accrue, but among two studies [50, 56] examining the effects of entire Wuqinxi exercise on attention concentration ability in middle-aged and elderly people, both 8-week training and 24-week training showed the same beneficial improvement on attention concentration ability. And two studies [58, 60] examined the therapeutic benefits of entire Wuqinxi exercise for female middle-aged and elderly patients with knee osteoarthritis that showed different dosing of Wuqinxi intervention, it is clearly seen whether six 60 min training sessions weekly for 24 weeks or six 60 min sessions for 16 weeks were helpful for treating knee osteoarthritis. Besides, only two included studies [23, 57] made a detailed discussion about one week of Wuqinxi learning phase, while the others only did comment on Wuqinxi practicing phase. It is indicated that Wuqinxi may be easy to learn and master whatever sex or different age levels, however, all of which may make it difficult to compare the results of these studies and introduce a risk of performance bias. Beyond the important similarities of movement and a focus on breath and mind to achieve Qigong states, there are other aspects that vary greatly within Wuqinxi training, including speed of execution, muscle groups used and range of motion, number of movements replication, and the number and style of animal movements used for intervention, all of which may provide differences in the physical and psychological outcomes. Therefore, modern advanced scientific and technological methods of interdisciplinary subjects should be adopted to explore “dosage” of Wuqinxi intervention in depth.

### 4.4. Outcome Measures

In this systematic review Wuqinxi was shown to be as effective as tai chi or Baduanjin exercise in slowing down memory decline or improving attention concentration ability and intelligence quotient in middle-aged and elderly people [37, 50, 56]. Besides, from the results we can acquire information that Wuqinxi had a beneficial regulation on immunologic function cross health middle-aged and elderly people [37, 45, 48, 49, 52]. However, the included studies did not provide sufficient evidence to prove the clinical efficacy of Wuqinxi. For example, Wuqinxi was shown to have positive effects on reducing subcutaneous adipose accumulation [40, 53, 55, 56]. It may be attributed to the low-to-moderate intensity levels of physical activity in Wuqinxi and its features of movements (e.g., flexion, bending, and rotation on a whole body). But scientific evidence reporting on the levels of physical activity (e.g., cardiorespiratory response, heart rate, and energy expenditure) in Wuqinxi is not yet determined. Although current Wuqinxi literatures use many standard outcome measures to evaluate the effects of Wuqinxi on cognitive function, immunologic function, and physical and psychological parameters, there are still some outcome measures that target the characteristics of the movements in Wuqinxi that are needed to be investigated. For example, movements in Wuqinxi involve both upper and lower limbs and emphasize symmetrical postures [14, 18, 19]; thus proprioception, bimanual coordination, fine motor control, and upper limb flexibility might be appropriate outcome measures in future studies. In addition to strengthening physical body, Wuqinxi involves regulating mind and breathing to cultivate an internal energy (i.e., a specific energy flow) [18, 19]. As a person practices the movements coordinated with the regulation of the mind and breathing, whether the internal energy will have a specific effect on improving neurophysiological parameters (e.g., neurotrophic factors, cerebral blood flow, brain-derived neurotrophic factor, insulin-like growth factor, and nerve growth factor, sleep quality, blood pressure, heart rate, sensorimotor function, forced expiratory flows, vital capacity, respiratory function, alveolar ventilation, blood flow and tissue volumes, and glomerular filtration rate) is unknown. Future researchers may consider adding the abovementioned outcome measures to determine whether nerve reaction ability, perceptual motor ability, and cardiovascular function as well as other system functions that can be improved by practicing Wuqinxi.

### 4.5. Randomization and Blinding

Among the 28 included studies, only sixteen were randomized [10, 22, 23, 38–40, 45–48, 51, 57–61].

And two trials [22, 59] described the method of randomization, while the others did not describe their methods of sequence generation or allocation concealment, suggesting that some declared RCTs may not be true RCTs. Meantime, in our included trials, there was no additional information about blinding of outcome assessment. Therefore, selection bias and detection bias were not ruled out owing to lack of detailed descriptions on randomization and blinding. Future studies examining the effects of Wuqinxi should clearly and concisely report the randomization process and focus on minimizing measurement bias by utilizing a model in which statisticians and assessors are blinded to the purpose of the clinical trial and subjects' group assignment. Another poor quality evidence is that, in most of these studies, Wuqinxi was preferentially provided to the intervention groups as a group therapeutic modality, whereas a matched number of social contact hours with coparticipants was not given to the control groups. Thus, performance bias might have existed in these trials and a placebo effect might have occurred in participants who benefited from participation in group activities and the contact with other persons.

### 4.6. Long-Term Effect and Adverse Events

Since follow-up assessments were rarely reported in the selected studies, a lack of follow-up might lead to difficulty in accounting for the long-term effect of Wuqinxi. Interestingly, the 24-week Wuqinxi program was shown to have protective effects on slowing down BMD loss [22, 59], which may be attributed to movement in Wuqinxi that emphasizes a high impact, weight-bearing movement for stimulating bone growth. Because bone rebuilding cycle can typically take up to 24 weeks [62], a 24-week or long-term Wuqinxi program could be more effective in attenuating BMD loss and should be examined in future studies. Thus investigators of future studies should examine the effects of Wuqinxi on health-related parameters by conducting RCTs or PPSs with a long-term follow-up assessment.

Qigong-related adverse physiological effects [9, 63–67], such as headache, dizziness or vertigo, distension of head, tinnitus, stuffiness in the chest and worsening shortness of breath, heart-pounding or palpitations, and muscular soreness or pain during or after Qigong training, have been noted in individuals who practice Qigong too long or too intensely, or failed to follow the principles of Qigong training or lack concentration and attention in Qigong training. In addition, Qigong-induced mental disorder (QIMD) is a recognized diagnosis in both the Chinese and American classification system of mental disorders [9, 65, 68, 69]. QIMD in the Qigong training refer to Qigong “deviation”, also known as “overrunning” of fire and entrance of demons, or deviation for short, is the serious negative somatic or mental reactions in the course of practicing Qigong. Deviation is represented by functional, psychological, emotional, or behavioral disorders that affect the practitioner's normal life or work and is unlikely to disappear spontaneously. Meanwhile, when applying Qigong and related therapies to special populations with severe psychiatric disorder, unwanted adverse reactions or Qigong “deviation” could have occurred. In addition, when Qigong and related therapies are implemented improper or overexuberant practice may induce further occurrence of psychotic episodes for people who have a family history or personal complications. On the other hand, people who do not suffer from psychosis but present with personality disturbance, eccentric conduct, and irrational thinking are not suitable for Qigong and related therapies, because they are at high risk of trigger-undesired Qi deviation (physical or more often emotional disorientation) during Qigong practice [9, 65]. There were no adverse effects of Wuqinxi reported in the included studies. The fundamental reason may be that all of different conditions of population in the included studies do not have severe psychiatric disorder or psychogenic family history and do not show personality disturbance, eccentric conduct, and irrational thinking. On the other hand, the Wuqinxi enjoys a long history and is esteemed as a typical, traditional Chinese* Dao Yin* practice. By mimicking the postures, movements, and bearing of the animals, along with their corresponding attitudes, practitioners will experience opening of the channels and network vessels, strengthening of the internal organs, and activation of the joints [9–14]. Thus Wuqinxi involves the combination of external motion with internal tranquility and the hard with the graceful, so as to attain tranquility in motion. Besides, while practicing the Wuqinxi, relax and hold the mind in* Dantian*. Breathe evenly and softly to unify the body and spirit. As a mind-body aerobic exercise, this form harmonizes firmness and softness in its design by embodying the fierceness of the tiger, the gentleness of the bird, and the agility of the ape [9]. Thus it is suitable for healthy people as well as patients with chronic diseases. And the people can select different routine of Wuqinxi (entirety or specific sections) to practice according to one's requires and conditions. It is generally recognized that it is difficult for Wuqinxi to result in adverse events or even QIMD. Wuqinxi as evaluated in this review generally seemed safe and well tolerated by health people and patients. However, most of studies were preceding papers without adequate reporting of essential details; the safety and adverse events still need to be clearly reported in future studies.

### 4.7. Practical Implications from the Current Evidence

Study findings in the existing literature indicated that Wuqinxi is thought to be beneficial for health in individuals with different health conditions, especially for people with chronic diseases. The positive effects of Wuqinxi among these populations may be attributed to the nature of Wuqinxi that emphasizes an integration of mind and body in practice. It is known that college students with mild depression disorder [57] and middle-aged and elderly adults who are experiencing cognitive function deficit [37, 50, 56] may have great difficulty in learning movement sequences. When they practiced the simple movements in Wuqinxi, these populations may have more confidence to make greater efforts during practice, leading to improved health-related parameters. Furthermore, an increased confidence will likely result in a greater enjoyment of the exercise. In addition, these populations with chronic diseases are typically sensitive to high-intensity and high-temperature exercise. Wuqinxi may be a suitable exercise modality that could be applied for assisting health professionals to treat disease-related symptoms. Our review provides comprehensive information about Wuqinxi training, which led to the suggestion for special populations to incorporate this beneficial exercise into a routine program. In this review a large number of databases were queried with relevant terms in title, abstract, and keywords. We are therefore confident that our search strategy has located all relevant data on the subject. Due to the language restriction to English and Chinese, a significant number of trials may have been excluded. Further limitations include the paucity and the suboptimal quality of primary data. In addition, we were unable to perform meta-analyses due to heterogeneity of study designs and outcome measures in the included studies. And we have not contacted relevant authors to identify unpublished or ongoing studies. Thus, publication bias might have existed in the included studies and the effect sizes of Wuqinxi might have been overestimated since positive trials are more likely to be published than negative trials. To provide further evidence for advocating Wuqinxi as a mind-body exercise to improve health status, studies with more vigorous procedures in randomization and blinding should be implemented to unravel the psychophysiological pathways suggested above. The adoption of a control group in future studies with a compatible “conventional therapy or attention placebo” is also important in order to rule out the effects of nonspecific factors. Compliance to the therapy should also be more thoroughly examined, as it is a major factor determining the health outcomes of the modalities. Thus researchers should pay attention to all possible strategies to prevent loss to follow-up for a certain period of time.

## 5. Conclusions

The results of the individual studies in this review indicate that Wuqinxi effectively improves health-related parameters in both healthy and clinical populations. However, there were significant limitations in the methodologies of many studies, which make it difficult to draw definitive conclusions. Designing better outcome measures will help answer not only the primary question of whether Wuqinxi works, but also the subsequent question of how Wuqinxi works. In addition, more studies should concentrate on same outcome measures, specific populations, and training intensity and duration for systematic evaluation of the effects of Wuqinxi. This can be done through a meta-analysis to provide stronger evidence on the effects of Wuqinxi. In order to enhance the validity and reliability of the research and provide more credible evidence for clinicians, the following improvements to research designs of RCTs should be considered: (1) blinding to randomization and implementation; (2) multisite RCTs with large sample sizes; (3) longer study periods to assess long-term effects of Wuqinxi on health condition; and (4) inclusion of more specific health outcomes or other disease profile.

## Figures and Tables

**Table 1:** Studies regarding effects of Wuqinxi exercise in healthy populations included in the analysis.

**Authors/ Year/ ** **Country**	**Study design/ ** **Blinding**	**Sample size**		**Mean age (year)/ Sex ** **(m/f)**	**Wuqinxi ** **practicing phases**	**Wuqinxi ** **Learning phase**	**Control intervention ** **design**	**Relevant ** **Outcomes**	**Results**	**Follow-up/ ** **Adverse events**	**Conclusion**
		**Wuqinxi**	**Control**								
Li-Ping Liang/ 2018/ China [37]	Historical cohort study/ Non-blinding	31 middle-aged and elderly people	30 Middle-aged and elderly people	Wuqinxi: 58.38±7.14 (16/15) Control: 57.65±8.27 (16/14)	Entire exercise: 30 mins/ session, 4 sessions/ week, at least 2 years	Not mentioned	unaltered lifestyle	1. Simplified version of graphic reasoning (Raven) test. 2. Cancellation test. 3. Self-report symptom inventory (SCL-90). 4. BFS mood scale. 5. Immunological index: CD3^+^, CD4^+^, CD8^+^, natural killer (NK) cells, immunoglobulins (IgG, IgA, IgM).	1. The scores of simplified version of graphic reasoning (Raven) test and cancellation test in Wuqinxi group were significantly higher than those in the control group (all *P* < 0.05). 2. The scores of somatic symptoms, depression, hostility, terror, paranoia and psychotic symptoms of SCL-90 in Wuqinxi group were significantly lower than those in the control group (all *P* < 0.05). 3. The scores of active, joyful and calm mood of BFS mood scale in Wuqinxi group were significantly higher than those in the control group (all *P* < 0.05), while the scores of thought, anger, excitement, depression and inactive in Wuqinxi group were significantly lower than those in the control group (all *P* < 0.05). 4. Immunological index of CD3^+^, CD4^+^, CD8^+^, natural killer (NK) cells in Wuqinxi group were significantly higher than those in the control group (all *P* < 0.05).	Not mentioned/ Not reported	Wuqinxi had effect on regulating and improving body immunity, cognitive function and psychological emotion in middle-aged and elderly people.

Feng Zhang et al. /2014/ China [10]	RCT/ Non-blinding	15 male volunteers	15 male volunteers	Age 41.3±3.5 (30/0)	Entire exercise: 5 times/week, 1 year	Not mentioned	Aerobic exercise (walking): 30 mins/ time, 5 times /week, 1 year	The average surface electromyography (ASEMG) in the process of flexion and extension was recorded and analyzed using DASYLab10.0 software, and the flexion extension ratio (FER) was calculated	The ASEMG in the process of flexion was lower than the ASEMG in the process of extension both before and after the 1 year exercise intervention on both groups of all volunteers. There was no significant difference in FER between the Wuqinxi group and control group before the 1 year Intervention. However, the FER of Wuqinxi group was lower than that of the control group after the 1 year intervention.	Not mentioned/ Not reported	Wuqinxi had effect on improving the function of the lumbosacral multifidus, and it thought to be an alternative method of reducing low back pain.

De-Sheng Chang et al. /2013/ China [38]	RCT/ Non-blinding	30 middle-aged and elderly people	Qiong *Yijinjing* group, Qigong *Liuzijue *group, Qigong *Baduanjin *group: 30 middle-aged and elderly people in each group	50≤age≤70 (16/104)	Entire exercise: 60 mins/session, at morning, 5 sessions/week, 12 weeks	Not mentioned	Qiong *Yijinjing *group, Qigong *Liuzijue *group, Qigong *Baduanjin *group: 60 mins/session, at morning, 5 sessions/ week12 weeks	Geriatric depression scale (GDS), self- rating anxiety scale (SAS), self-rated health measurement scale version (SRHMS)	The four kinds of Qigong exercises had positive effects on psychological emotion of middle-aged and elderly people, but their emphasis was different. In terms of improving depression: Qiong *Yijinjing* group > Qigong *Liuzijue* group > Qigong *Baduanjin *group > Qigong *Wuqinxi* group. In terms of improving anxiety: Qiong *Liuzijue* group > Qigong *Wuqinxi* group > Qigong *Yijinjing* group > Qigong *Baduanjin* group. In terms of improving self-test health: Qiong *Baduanjin* group > Qigong *Wuqinxi *group > Qigong *Liuzijue* group > Qigong *Yijinjing* group.	Not mentioned/ Not reported	The four kinds of Qigong exercises showed good health preservation value, and had a positive impact on regulating and improving middle-aged and elderly people's mental state. Because of the emphasis of Qigong exercise, middle-aged and elderly people who are interested in Qigong exercises can choose a Qigong exercise suitable for themselves according to their specific circumstances and hobbies.

Gang Qin/2012/ China [39]	RCT/ Non-blinding	10 healthy college students	10 healthy college students	21≤age≤22 (unspecified gender ratio)	Entire exercise: 60 mins/session, 5 sessions/week, 20 weeks	Not mentioned	unaltered lifestyle	Cardiac function index and vascular function index: pulse rate (HR), stroke volume (SV), cardiac index (CI), left ventricular pump power effective (VPE), mean systolic pressure (MSP), mean diastolic pressure (MDP), mean arterial pressure (MAP), vascular compliance (AC), total peripheral resistance (TR), stroke index (SI), high density lipoprotein cholesterol (HDL-C), low density lipoprotein cholesterol (LDL-C), vascular elastic dilatation coefficient (ETK).	The cardiac vascular main index of the control group had no significant changes. SV, CI, VPE, the HDL increased significantly, HR, MSP, MDP, MAP, the TR dropped obviously in Wuqinxi group.	Not mentioned/ Not reported	Wuqinxi was thought to have a positive impact on cardiovascular function of healthy college students.

Lei Yu et al./ 2012/ China [40]	RCT/ Non-blinding	60 Middle-aged and elderly people	60 Middle-aged and elderly people	Age: 68.29±2.17 (unspecified gender ratio)	Entire exercise: 3 times/week, 1 year	Not mentioned	conventional exercise	1. Physiological function index: systolic pressure (SP), diastolic pressure (DP), heart rate (HR), vital capacity (VC). 2. Physical quality index: back strength, grip strength, trunk bending forward, sit-up, push-up, vertical jump, agility test. 3. Biochemical index: hemoglobin, albumin, glucose, triglyceride, cholesterol.	Before exercise, there was no significant difference in physiological function index and physical quality index, biochemical index between Wuqinxi group and control group. After the 1-year intervention period, all physiological function index and physical quality index, biochemical index in Wuqinxi group were significantly improved. Whereas these positive changes were not observed in the control group.	Not mentioned/ Not reported	Wuqinxi was thought to have better effect on improving physiological function and physical quality of middle-aged and elderly people.

Li-Mei Duan/ 2012/China [41]	PPS/ Non-blinding	60 elderly people	-* *-* *-	Age: 64.0±3.4 (28/32)	Entire exercise: 45 mins/session, at morning, 6 sessions/week, 16 weeks	Not mentioned	-* *-* *-	1. Serum antioxidant index: malondialdehyde (MDA), superoxide dismutase (SOD), catalase (CAT), glutathione (GSH), glutathione peroxidase (GSH- Px), glutathione reductase (GR). 2. Intestine lactobacillus.	Compared with pre-exercise, the concentration of MDA decreased significantly, the concentration of GSH, the activity of SOD, CAT, GSH-Px and GR and the quantity of intestine lactobacillus increased significantly after 8 weeks and 16 weeks.	Not mentioned/ Not reported	Wuqinxi exercise showed effects of producing SOD, GSH and reducing substance to enhance antioxidant capacity and increase quantity of intestine lactobacillus.

Yu-Hua Bai et al. /2012/ China [42]	Historical cohort study/ Non-blinding	15 healthy middle-aged and elderly people	15 healthy middle-aged and elderly people	Wuqinxi: 62.63±3.07 (7/8) Control: 62.71±2.93 (8/7)	Entire exercise: 5 times/week, at least 1 year	Not mentioned	Aerobic exercise (walking): at least 30 mins/time, 5 times/ week, 1 year	The average surface electromyography (ASEMG) in the process of flexion and extension was recorded and analyzed using Biovision surface electromyography test system software, and the flexion extension ratio (FER) was calculated.	The ASEMG of the process of flexion was less than that of process of extension in two group. In addition, the FER of the Wuqinxi group was significantly lower than that of the control group.	Not mentioned/ Not reported	Wuqinxi was thought to have positive improvement on the function of the lumbosacral multifidus in the middle-aged and elderly people so as to reduce low back pain.

Quan-Xi Sang/ 2011/China [43]	PPS/ Non-blinding	55 obese older people	-* *-* *-	Male: 55≤age≤61 Female: 50≤age≤60 (33/22)	Entire exercise: 120 mins/session / day, 48 weeks	Not mentioned	-* *-* *-	1. Serum antioxidant index: malondialdehyde (MDA), superoxide dismutase (SOD), catalase (CAT), peroxidase (GSH- Px). 2. Lipid peroxidation index: total cholesterol (TC), triglyceride (TG), high density lipoprotein cholesterol (HDL-C), low density lipoprotein cholesterol (LDL-C). 3. Intestine lactobacillus: bacillus acidophilus, lactobacillus casei and bacillus bifidus.	48 weeks of Wuqinxi could markedly enhanced blood antioxidant enzymes activities, lipid peroxidation and intestine probiotics count in obese older people.	Not mentioned/ Not reported	Wuqinxi was thought to be beneficial for obese older people.

Yan-Yang Chen/ 2011/China [44]	PPS/ Non-blinding	64 healthy older people	-* *-* *-	Age: 60.3±5.61 (23/41)	Entire exercise: 60 mins/session/ day, at morning, 30 days	Not mentioned	-* *-* *-	Malondialdehyde (MDA), total cholesterol (TC), triglyceride (TG), high density lipoprotein cholesterol (HDL-C), low density lipoprotein cholesterol (LDL-C), superoxide dismutase (SOD), catalase (CAT)	30 days of Wuqinxi could significantly reduce the serum levels of TC, TG and LDL-C, while increased the serum level of HDL-C and activities of SOD and CAT.	Not mentioned/ Not reported	30 days of Wuqinxi exercise can decreased blood lipids levels and oxidative injury in older people. Wuqinxi was thought to be useful for older peoples' health.

Ding-Hai Yu et al. /2008/ China [45]	RCT/ Non-blinding	50 middle-aged and elderly people	50 middle-aged and elderly people	Male: 61.6±3.8 Female: 58.5±4.1 (30/70) Wuqinxi: (12/34) Control: (15/35)	Entire exercise: 26-39 mins/session, at least 4 sessions/ week, 24 weeks	Not mentioned	unaltered lifestyle	Plasma NK cell activity	After 24-week of Wuqinxi, the plasma NK cell activity of middle-aged and elderly people in the Wuqinxi group was obviously higher than that before exercise, while no obvious change had appeared in the control group.	Not mentioned/ Not reported	Wuqinxi was a middle or low intensity aerobic exercise, it was thought to have an effect on improving state of mind, increasing the activity of NK cells and effectively adjusting the balance of immune ability in middle-aged and elderly people.

Ding-Hai Yu /2008/ China [46]	RCT/ Non-blinding	50 middle-aged and elderly people	50 middle-aged and elderly people	Male: 61.6±3.8 Female: 58.5±4.1 (30/70) Wuqinxi: (15/35) Control: (15/35)	Entire exercise: 45 mins/session, at least 4 sessions/ week, 24 weeks	Not mentioned	unaltered lifestyle	Cholesterol (TC), triglyceride (TG), high density lipoprotein cholesterol (HDL-C), low density lipoprotein cholesterol (LDL-C)	After 12-week of Wuqinxi, the TG of Wuqinxi group was significantly lower than that before Wuqinxi exercise (*P*<0.01), the TC, HDL-C, LDL-C of female in Wuqinxi group was significantly higher than that before Wuqinxi exercise (all *P*<0.05). After 24-week of Wuqinxi exercise, remarkable decreased for TG and significant increased for LDL-C were observed in the Wuqinxi group (all *P*<0.05).	Not mentioned/ Not reported	24-week of Wuqinxi showed a certain effect on improving lipid metabolism in the middle-aged and elderly people.

Han-Xiao Zhu/2008/China [47]	RCT/ Non-blinding	45 female elderly people	30 female elderly people	Wuqinxi: 62.8±1.4 (0/45) Control: 63.4±1.8 (0/30)	Entire exercise: 45 mins/session/ day, 16 weeks	Not mentioned	unaltered lifestyle	Malondialdehyde (MDA), superoxide dismutase (SOD), estradiol (E2), cardiac functional capacity (FC) test, blood pressure (BP), heart rate (HR), vital capacity (VC), reaction time, exercise time, time for close eyes one-foot balance.	After Wuqinxi exercising, the activity of SOD and the level of estradiol in the Wuqinxi group were significantly higher than those before Wuqinxi exercising (all *P*<0.05), the content of MDA in the Wuqinxi group was significantly higher than that before Wuqinxi exercising, but it was remarkably lower than that in the control group. In addition, time for close eyes one-foot balance showed increase in the Wuqinxi group after exercising, which was significantly longer than that in the control group (*P* <0.05). At the same time, significant decresed were observed in exercise time of Wuqinxi group after exercising (*P* <0.05).	Not mentioned/ Not reported	Wuqinxi was thought to have an effect on improving the activity of peripheral blood SOD, delaying the damage of free radical, increasing the level of sex hormone, and enhancing the ability of nerve reaction and balance in the female elderly people.

Ding-Hai Yu /2007/ China [48]	RCT/ Non-blinding	50 middle-aged and elderly people	50 middle-aged and elderly people	Male: 61.6±3.8 Female: 58.5±4.1 (30/70) Wuqinxi: (12/34) Control: (15/35)	Entire exercise: 45 mins/session, at least 4 sessions/ week, 24 weeks	Not mentioned	unaltered lifestyle	Immunological index (Peripheral blood T-cell subsets): CD_4_^+^, CD_8_^+^, CD_3_^+^ and the ratio of CD_4_^+^/ CD_8_^+^.	After 24-week of Wuqinxi, the CD_8_^+^ of male subjects in Wuqinxi group was significantly lower than that before Wuqinxi exercise, and the ratio of CD_4_^+^/ CD_8_^+^ of male subjects in Wuqinxi group was remarkably increased after exercise. Meanwhile, Wuqinxi group showed the CD_3_^+^ increase in female subjects, a decreased in CD_8_^+^ was observed in female subjects of Wuqinxi group after exercise. Thus, the ratio of CD_4_^+^/ CD_8_^+^ of female subjects in Wuqinxi group was also remarkablely increased after exercising.	Not mentioned/ Not reported	24-week of Wuqinxi showed a positive effect on the peripheral blood T lymphocytes of middle-aged and elderly people, and it may be better regulated the immune balance of middle-aged and elderly people.

Jing-Mei Wu et al. /2006/ China [49]	PPS/ Non-blinding	50 middle-aged and elderly people	-* *-* *-	Age: 59.2±3 (15/35)	Entire exercise: 45 mins/session, 4 sessions/week, 12 weeks	Not mentioned	-* *-* *-	Immunological index (Peripheral bl-ood T-cell subsets): CD_4_^+^, CD_8_^+^, CD_3_^+^ and ratio of CD_4_^+^/ CD_8_^+^.	Wuqinxi showed a positive effect on improving the immunity of middle-aged and elderly people, and the immunity ability of female practitioners grew even faster. Furthermore the extents of effect varied according to different age groups and the practitioners between 60 and 69 improved faster in the ability of immunity.	Not mentioned/ Not reported	Practicing Wuqinxi may be affected the distribution of the outer sub-group of T-cell of middle-aged and elderly people. And it is good for the improvement of the immunity of middle-aged and elderly people.

Xiu-Ying Chen et al. / 2006/ China [50]	PPS/ Non-blinding	100 middle-aged and elderly people	-* *-* *-	50≤age≤70 (42/58)	Entire exercise: 45 mins/session, 7 sessions/week, 24 weeks	Not mentioned	-* *-* *-	Attention concentration testing apparatus (ACTA)	After 24-week of Wuqinxi, attention concentration ability was significantly improved in middle-aged and elderly, the improvement degree of female volunteers was higher than that of male volunteers and the improvement degree of attention ability in the 61~70 year old middle-aged and elderly volunteers is better than that in the 50~60 year old middle-aged and elderly volunteers.	Not mentioned/ Not reported	Exercising Wuqinxi showed an effect on improving the middle-aged and elderly people's attention concentration ability.

Han-Xiao Zhu/2006/China [51]	RCT/ Non-blinding	45 female elderly people	30 female elderly people	61≤age≤65 (0/75)	Entire exercise: 45 mins/session/ day, at morning, 16 weeks	Not mentioned	unaltered lifestyle	Malondialdehyde (MDA), superoxide dismutase (SOD), estradiol (E2), cardiac functional capacity (FC) test, blood pressure (BP), heart rate (HR), vital capacity (VC), reaction time, exercise time, time for close eyes one-foot balance	After 16-week of Wuqinxi exercise, the activity of SOD of Wuqinxi group was increased, meanwhile, although the MDA was also increased, it was obvious lower than that of control group. The E2 of the female in the Wuqinxi group was increased. The FC had no obvious change after 16-week exercise, but it was prone to increase. The reaction time of Wuqinxi group had no significant change after Wuqinxi exercise, the exercise time of Wuqinxi group was obviously shorted, the time for close eyes one-foot balance of Wuqinxi group was markedly prolonged, which was obviously higher than that of control group.	Not mentioned/ Not reported	Wuqinxi was thought to be effectively slowed the process of ageing by increasing serum SOD activity, delaying the free radical injury increased with ageing, improving the level of sexual hormone, and enhancing feeling and balance ability.

Ding-Hai Yu et al. /2005/ China [52]	PPS/ Non-blinding	53 middle-aged and elderly people	-* *-* *-	50≤age≤67 (15/38)	Entire exercise: 26~39 mins/ session, at least 4 sessions/week, 12 weeks	Not mentioned	-* *-* *-	Plasma NK cell activity	After 12-week of Wuqinxi exercise, the plasma NK cell activity of Wuqinxi group showed obviously higher than that before exercise, the increased degree of female middle-aged and elderly people was higher than that of male middle-aged and elderly people.	Not mentioned/ Not reported	Wuqinxi was a middle or low intensity aerobic exercise, it was thought to have better effect on regulating the immunologic balance of middle-aged and elderly people.

Yong-Sheng Cui et al. /2004/ China [53]	PPS/ Non-blinding	200 female middle-aged and elderly people	-* *-* *-	55≤age≤70 (0/200)	Entire exercise: 60 mins/session, 3 sessions/week, 12 weeks	Not mentioned	-* *-* *-	1. Physiological index: height, weight, waistline, hipline, thickness of upper arm and subscapular skin fold, pulse, systolic pressure, diastolic pressure, vital capacity, bone density, grip strength, trunk bending forward, time for close eyes one-foot balance, reaction time, maximal oxygen intake, body fat rate, waist/ hip ratio, body mass index, percentage of forced expiratory volume in first second to forced vital capacity. 2. Psychological index: self-rated health measurement scale version (SRHMS).	Female middle-aged and elderly people's blood pressure, pulse, waist/ hip ratio, grip strength and vital capacity have had obvious improvement after the exercise of Wuqinxi 12 weeks later, at the same time, an evident effect on mentality follows.	Not mentioned/ Not reported	The layout of Wuqinxi exercise was thought to be good effect on middle-aged and elderly females' body and mind.

Ding-Hai Yu et al. /2004/ China [54]	PPS/ Non-blinding	71 female middle-aged and elderly people	-* *-* *-	Age: 55.1±5.1 (0/71)	Entire exercise: 60 mins/session, 3 sessions/week, 12 weeks	Not mentioned	-* *-* *-	Height, weight, waistline, hipline, thickness of upper arm and subscapular skin fold, pulse, systolic pressure, diastolic pressure, vital capacity, bone density, grip strength, trunk bending forward, time for close eyes one-foot balance, reaction time, maximal oxygen intake, body fat rate, waist/ hip ratio, body mass index, percentage of forced expiratory volume in first second to forced vital capacity. 2. Psychological index: self-rated health measurement scale version (SRHMS).	Female middle-aged and elderly people's waistline, waist/ hip ratio, systolic pressure, diastolic pressure have had obvious decreased after the 12-week of Wuqinxi, at the same time, an evident improvement effect on vital capacity and grip strength after exercise.	Not mentioned/ Not reported	12-week of Wuqinxi had positive effects on body shape, physical function and physical quality of female middle-aged and elderly people.

Ding-Hai Yu et al. /2003/ China [55]	PPS/ Non-blinding	85 healthy middle-aged and elderly people	-* *-* *-	Male: 64.4±4.9 Female: 55.1±5.1 (14/71)	Entire exercise: 7 times/week, 8 weeks	Not mentioned	-* *-* *-	1. Body shape index: height, weight, waistline, hipline, body fat rate, waist/ hip ratio, body mass index thickness of upper arm and subscapular skin fold. 2. Physical function index: pulse, systolic pressure, diastolic pressure, pulse pressure, vital capacity, bone density, percentage of forced expiratory volume in first second to forced vital capacity. 3. Physical quality index: grip strength, trunk bending forward, time for close eyes one-foot balance, reaction time, maximal oxygen intake. 4. Psychological index: self-rated health measurement scale version (SRHMS).	Wuqinxi = had a positive effect on the body shape, physical function and physical quality of the volunteers, and also had a better effect on improving and adjusting the mental state of the volunteers.	Not mentioned/ Not reported	Wuqinxi not only accorded with the principle of "Three regulations (body, breath, mind)" of traditional Qigong and the law of modern human body kinematics, but also can effectively improve the physical and psychological health of the middle-aged and elderly exercisers.

Jia-Duo Wu et al. /2003/ China [56]	PPS/ Non-blinding	85 healthy middle-aged and elderly people	-* *-* *-	Male: 64.4±4.9 Female: 55.1±5.1 (14/71)	Entire exercise: 7 times/week, 8 weeks	Not mentioned	-* *-* *-	1. Self-rated health measurement scale version (SRHMS). 2. Attention concentration testing apparatus (ACTA). 3. Perceived- exercise-effect rating scale (PEERS).	After 8-week of Wuqinxi exercise, the physical and mental function of the volunteers, especially the mental health sub scale, showed a preliminary change, indicating that the wuqinxi had a certain effect on improving and regulating the mental state of the individual. The improvement of body symptoms, positive emotions and error time indicators of attention concentration testing in female was better than that in male after exercise. Error time indicators of attention concentration testing in 55 years old volunteers were less than that in 56~65 years old volunteers. Wuqinxi exercise was accepted by most volunteers.	Not mentioned/ Not reported	Wuqinxi was thought to have certain effects on stabilizing mood, regulating physical and psychological state, and improving the quality of life in middle-aged and elderly people.

**Table 2:** Studies regarding effects of Wuqinxi exercise in clinical populations included in the analysis.

**Authors/Year/ Country**	**Study design/ Blinding**	**Sample size**		**Mean age (year) Sex (m/f)**	**Wuqinxi Practicing phases**	**Wuqinxi Learning phase**	**Control Intervention design**	**Relevant Outcomes measured**	**Results**	**Follow-up/ Adverse events**	**Conclusion**
		**Wuqinxi**	**Control**								
Xiang Cheng et al. /2016/ China [57]	RCT/ Non-blinding	15 college students with mild depression; 14 healthy college students	15 college students with mild depression; 14 healthy college students	Wuqinxi: 1. Mild depression: 21.1±1.4 (7/8) 2. Healthy: 21.2±1.2 (7/7) Control: 1. Mild depression: 21.0±1.6 (8/7) 2. Healthy: 20.9±1.6 (7/7)	Entire exercise: 40~60 mins/ session at 6:00~8:00 or 3 h before bedtime, 3 sessions/week, 12 weeks	1 week of learning under the guidance of professional teachers before intervention	unaltered lifestyle	1. BECK depression self-reported questionnaire (BDI). 2. Hamilton depression rating scale (HAMD). 3. The metabolic parameters of 1H- MRS in the prefrontal cortex and hippocampus: NAA/ Cr, Cho/ Cr, NAA/ Cho, Cho/ NAA.	Before the intervention, the scores of BDI and HAMD in the mild depression group were significantly higher than that in the control group ( all *P*<0.01), and were lowered obviously after the 12-week intervention (all *P*<0.01). Compared with the control group, 1H-MRS in the mild depression group before intervention showed significantly increased NAA/Cr value in the left prefrontal cortex, Cho/Cr value in the bilateral hippocampus and the left frontal lobe, and Cho/Cr value of the left hippocampus and right frontal lobe (*P*<0.05) with significantly lowered NAA/Cho value in the	Not mentioned/ Not reported	Exercising Wuqinxi was thought to reduce depression scale scores in college students with mild depression and improve the metabolic index (NAA/Cr and Cho/Cr values) in the prefrontal cortex and the hippocampus.

									bilateral prefrontal and Cho/ NAA value in the right hippocampus (*P*<0.05). After 12 weeks of intervention, NAA/Cr value in the bilateral hippocampus and the NAA/Cho value in the right hippocampus were significantly lowered (*P*<0.05), and NAA/Cho value in the right prefrontal and Cho/NAA value in the right hippocampus were significantly increased (*P*<0.05) in the mild depression group. Before the intervention, Pearson correlation analysis showed that the scores of HAMD and BDI were positively correlated with Cho/Cr value in the hippocampus and NAA/Cr value in prefrontal lobe (*P*<0.01) and inversely with NAA/Cho in prefrontal lobe and Cho/NAA value in the hippocampus (*P*<0.05). After the intervention, the scores of HAMD and BDI were positively correlated with NAA/Cr value in the hippocampus and Cho/Cr value in the left hippocampus (*P*<0.05).		.

Mao-Rong Shen et al. /2014/ China [22]	RCT/Non-blinding	100 elderly patients with senile osteoporosis	100 elderly patients with senile osteoporosis	Wuqinxi: 68.69±5.18 (46/54) Control: 69.25±5.27 (45/55)	Entire exercise: 45 mins/session, at morning, 6 sessions/week, 24 weeks	Not mentioned	Treating with ibuprofen sustained release capsules (Fenbid) and calcium carbonate and vitamin D3 tablets, 1tablet/time, 2 times/day	1. Bone metabolism index: serum osteocalcin (BGP), alkaline phosphatase (ALP), level of pyridinoline (PYD). 2. Visual analogue scale (VAS): low back pain score.	24 weeks later, low back pain score of the cases in the Wuqinxi group was obviously increased, compared to that of the control group with a significant difference (*P*<0.05). Meanwhile, though there was no significant difference between the two groups' figures related to bone metabolism, patients in the control group showed an increase in serum osteocalcin (BGP) and alkaline phosphatase (ALP) alongside with a reduction in the level of pyridinoline (PYD).	Not mentioned/ Not reported	Practicing Wuqinxi was positive to the bone metabolism of the senile osteoporosis patients and can effectively relieved and improved the low back pain syndromes of the senile osteoporosis patients. To a certain degree, this exercise can also increase bone formation and decreased bone resorption. It was an effective way to prevent the senile osteoporosis disease and deserved carrying out in communities.

Ping Tu et al. /2014/ China [58]	RCT/Non-blinding	20 female patients with knee osteoarthritis	20 female patients with knee osteoarthritis	Age ≥ 50 years (0/40)	Entire exercise: 60 mins/session, 6 sessions/week, 16 weeks	Not mentioned	Qigong *zhanzhuang *(standing exercise): 10mins/session, 3 sessions/ time/day, at morning, noon, evening, 6 times/week, 4 months	1. The peak torque (PT) and total work (TW) of the affected knee were obtained by the isokinetic testing system. 2. The level of pain, stiffness, dysfunction measured by The Western Ontario and McMaster Universities (WOMAC) Osteoarthritis Index Scale.	After intervention, the peak torque (PT) and total work (TW) in both groups were improved compared with those of pre- intervention, and the Wuqinxi group indicated a better improvement in PT and TW. The WOMAC scores went down in both groups after intervention, and the score of pain, dysfunction and the total score were lower in the Wuqinxi group with a statistical significance.	Not mentioned/ Not reported	Wuqinxi and Zhanzhuang were thought to improve the quadriceps strength of female patients with KOA and reduce the effect of pain, stiffness and dysfunction and so on. Wuqinxi was considered to have a smooth and comprehensive influence on the knee's flexor and extensor strength of female patients with KOA when compared with Zhanzhuang and it was helpful for relieving pain and reducing dysfunction.

Mao-Rong Shen et al. /2013/ China [59]	RCT/Non-blinding	100 elderly patients with senile osteoporosis	100 elderly patients with senile osteoporosis	Wuqinxi: 68.69±5.18 (46/54) Control: 69.25±5.27 (45/55)	Entire exercise: 45 mins/session, at morning, 6 sessions/week, 24 weeks	Not mentioned	Treated with ibuprofen sustained release capsules (Fenbid) and calcium carbonate and vitamin D3 tablets, 1tablet/time, 2 times/day	1. The bone mineral density (BMD) of lumbar vertebrae. 2. Visual analogue scale (VAS): low back pain score.	After 24-week treatment, BMD of the lumbar vertebrae in Wuqinxi group was higher than that in control group significantly (*P*<0.05). And the low back pain scores of the two groups were significantly different (*P*<0.05).	Not mentioned/ Not reported	Practicing Wuqinxi increased BMD of the lumbar vertebrae in senile patients with osteoporosis, and decreased their low back pain. It was an effective way to prevent and cure primary osteoporosis, and can be applied in communities.

Hai-Ming Liu /2012/ China [23]	RCT/Non-blinding	22 elderly patients with metabolic syndrome	18 elderly patients with metabolic syndrome	60≤age≤75 Wuqinxi (10/12) Control (9/9)	Entire exercise: 60 mins/session, at morning, 6 sessions/week, 24 weeks	1 week of learning under the guidance of professional teachers before intervention	unaltered lifestyle	1. The vascular risk factors: body mass index (BMI), fasting blood gluco (FBG), total cholesterol (TC), triglyceride (TG), high density lipoprotein cholesterol (HDL-C), low density lipoprotein cholesterol (LDL-C). 2. The neuropsychological index: mini-mental state examination (MMSE), Montreal cognitive assessment (MOCA), verbal fluency test (VFT), trail making test (TMT), Hamilton depression rating scale (HAMD).	Compared with control group and pre-exercise of Wuqinxi group, the vascular risk factors including BMI, FBG, TC, TG, LDL and HDL and the neuropsychological index including MMSE, visual space and executive ability, naming, attention, delayed recalling, orientation, total MOCA, HAMD and VFT had significantly beneficial changes after exercise in Wuqinxi group. There was significant negative correlation between WC, SBP, FBG, TC, TG, LDL and cognitive function, significant positive correlation between HDL and cognitive function.	Not mentioned/ Not reported	The vascular risk factors and the cognitive function of elderly patients with metabolic syndrome were improved after Wuqinxi exercise. The positive effect of Wuqinxi on vascular risk factors was likely to be the physiological mechanism that Wuqinxi could improve the cognitive function of elderly patients with metabolic syndrome.

Bing-Wu Tian /2012/ China [60]	RCT/Non-blinding	20 female middle-aged and elderly patients with knee osteoarthritis	Control ①: 20 female middle-aged and elderly patients with knee osteoarthritis. Control ②: 20 healthy middle-aged and elderly females	60≤age≤70 (0/60)	Entire exercise: 60 mins/session, 6 sessions/week, 24 weeks	Not mentioned	unaltered lifestyle	1. Body weight body mass index (BMI), percentage of body fat (PBF), lower extremity response latencies, vertical jump height, and no pain range of motion (ROM) of the affected limb, proprioception and balance function of the affected limb. 2. The Western Ontario and McMaster Universities (WOMAC) Osteoarthritis Index Scale.	The body weight, BMI and the percentage of body fat of KOA female middle-aged and elderly patients can be reduced by long-term Wuqinxi exercise, which could also effectively improved the reaction time of lower limbs, the vertical jump height and the painless ROM of knee-joint without pain and, reduced the active and passive 30° and 60° angle reconstruction error of KOA patients, availably improved the sagittal and lateral stable ability, enhance their stable limit, and decreased the fall risk, clinical medical treatment should effectively relieved pain, spasticity, action limited and improve their body function.	Not mentioned/ Not reported	It was good for middle-aged and elderly patients with KOA to practice Wuqinxi in a long term, which can effectively improve the proprioceptive function, dynamic and static balance of knee and reduced their risk of falls. The effect may be related to decrease of body weight and percentage of body fat and the increase of strength, reactive speed and ROM of the affective knee. Clinical treatment can effectively alleviate the KOA patients with symptoms such as pain, stiffness and behavior limited, but the clinical treatment with Wuqinxi practice seems to be able to make the effect more pronounced and lasting.

Zhao-Wei Li et al. /2009/ China [61]	RCT/Non-blinding	33 patients with dyslipidemia	33 patients with dyslipidemia	Wuqinxi: 58.67±20.43 (19/14) Control: 56.47±24.15 (21/12)	Entire exercise: 30 mins/session/ day, 16 weeks	Not mentioned	Aerobic exercise (jogging): 30 mins/session/ day, 16 weeks	Cholesterol (TC), triglyceride (TG), high density lipoprotein cholesterol (HDL-C), low density lipoprotein cholesterol (LDL-C)	After 16 weeks of Wuqinxi, TC, TG and LDL-C of Wuqinxi group declined remarkably (all *P*<0.01) and HDL-C of this group rose evidently (*P*<0.05). TC, TG and LDL-C of Wuqinxi group changed much more dramatically than that of the control group (*P*<0.05). In addition, the rate of reaching standard in the Wuqinxi group was higher than that in the control group (*P*<0.05).	Not mentioned/ Not reported	Wuqinxi showed effective for patients with dyslipidemia.
